# The effect of e-learning on point-of-care ultrasound education in novices

**DOI:** 10.1080/10872981.2022.2152522

**Published:** 2022-11-26

**Authors:** Wan-Ching Lien, Phone Lin, Chih-Heng Chang, Meng-Che Wu, Cheng-Yi Wu

**Affiliations:** a Department of Emergency Medicine, National Taiwan University Hospital, Taipei, Taiwan; b Department of Emergency Medicine, College of Medicine, National Taiwan University, Taipei, Taiwan; c Department of Computer Science & Information Engineering, National Taiwan University, Taipei, Taiwan; d Diversion of Critical Care Medicine, Department of Emergency and Critical Care Medicine, Fu-Jen Catholic University Hospital, New Taipei, Taiwan

**Keywords:** e-learning, point-of-care ultrasound (PoCUS), the first postgraduate year (PGY-1), focused assessment of sonography for trauma (FAST), resident

## Abstract

**Background:**

Current studies assessed the learning efficacy of e-learning in ultrasound (US) training using questionnaires, or simulation in well-controlled conditions. This study investigates the effect of e-learning on the clinical US performance of the first postgraduate year (PGY-1) residents.

**Methods:**

In this prospective observational study, we enrolled PGY-1 and second postgraduate year (PGY-2) residents. The e-learning was introduced on the first day and each PGY-1 was authorized to access the e-learning platform. The point-of-care ultrasound (PoCUS) curriculum for the focused assessment of sonography for trauma (FAST) was conducted on the 7^th^ day for PGY-1 and the objective structured clinical examination (OSCE) followed. The PGY-2 received bedside one-to-one random learning before the study and did not have the authorization to access the e-learning. The FAST examinations performed by the PGY-1 and PGY-2 were collected on the 30th day. The clinical FAST performance was assessed by the instructor not involved in the curriculum and blinded to the use of e-learning, including numbers, image quality, and diagnostic accuracy between PGY-1 e-learning users, non-users, and the PGY-2.

**Results:**

One hundred and seventy PGY-1 with 736 FAST examinations and 53 PGY-2 residents with 134 examinations were included. Seventy PGY-1 used e-learning with a median time spent of 13.2 mins (IQR, 6.5–21.1 mins) at the first access. The PGY-2 had more PoCUS experience than the PGY-1, however, the 70 e-learning users performed more FAST examinations than the PGY-2 (median [IQR], 4 [2–6] vs. 2 [1–3], p = 0.0004) and had better image quality than the PGY-2 (3 [3–3.2] vs. 3 [2.7–3], p = 0.044). There were no significant differences in the diagnostic accuracy between the PGY-1 and PGY-2.

**Conclusions:**

E-learning has a positive effect on US learning. The PGY-1 users had comparable performance with the PGY-2 and even better image acquisition although the PGY-2 had more PoCUS experience.

**Trial registration:**

NCT03738033 at ClinicalTrials.gov.

## Introduction

The American College of Emergency Physicians states that point-of-care ultrasound (PoCUS) is an essential skill in emergency practice and suggests 12 core applications [1]. The focused assessment of sonography for trauma (FAST) is a fundamental application, not only for trauma victims but also for providing valuable information for non-trauma patients [2,3].

PoCUS can be utilized effectively with proper training. The Accreditation Council for Graduate Medical Education has recommended that emergency residency training programs provide residents with competency in PoCUS [4]. However, it usually takes at least 4 weeks for the residents to finish an education program [5]. An emergency resident has to perform at least 50 sonographic examinations for the FAST application to have recognizable image acquisition [6], which could be challenging due to the time constraints and inflexible schedules of trainees in PoCUS training [7].

Traditionally, didactics and bedside hands-on teaching are adopted for ultrasound (US) education. Electronic learning (e-learning) provides more flexibility compared to traditional teaching [8]. E-learning is the product of internet technologies to enhance knowledge and performance, involving the use of online multimedia and/or interactive programs. It is considered beneficial as an adjunct to supplement didactic learning [9,10].

Most of the current studies assessed the learning efficacy of e-learning in US training using questionnaires, or simulation in well-controlled conditions [11–13]. To our knowledge, the real effect of e-learning on clinical PoCUS performance is not evaluated thoroughly [11,14].

After finishing the medical school program in Taiwan, the graduates participate in the one-year postgraduate general medicine training before they start residency in a specialty. One-month emergency medicine training is included in the first postgraduate year (PGY-1) program in which a PoCUS curriculum was implemented. In our previous work [15], we designed an interactive e-learning platform for the PGY-1, being a part of PoCUS training. This study aims to investigate the effect of e-learning on the clinical FAST performance of PGY-1, compared with the second post-graduate year (PGY-2) residents.

## Materials and methods

### Study setting

This prospective observational study was conducted at the Emergency Department (ED) of the National Taiwan University Hospital between July 2015 and October 2017. The study protocol was approved by the institutional review board of the hospital (201412004RIND) and registered at ClinicalTrials.gov (NCT03738033). Written informed consent was obtained from each PGY-1 and PGY-2 resident.

Two high-resolution US machines (SSA-550A, SSA-660A, Canon, Japan) equipped with 2–5 MHz curvilinear transducers were ready for use.

### Study design

Every PGY-1 resident went through the same program for PoCUS training:

1. On the 1st day: We introduced the PoCUS training schedule and the e-learning platform. Each resident willing to attend this study was assigned unique identification and authorized to access the platform.
2. On the 7th day: The FAST curriculum included 30-min didactics and 2-hour hands-on training on a healthy volunteer. The instructors were board-certified by the Taiwan Society of Emergency Medicine. The ratio of the instructors to the residents was 1:5. The instructor who was blinded to the use of e-learning was responsible for the objective structured clinical examination (OSCE) for FAST application after the curriculum.

The OSCE included basic questions for image acquisition and interpretation with points for technique, image quality, and correct interpretation of the anatomy of the FAST application (Supplementary file 1). The instructor gave global rating scores using a Likert 5-point scale (1, unsatisfactory; 2, needs improvement; 3, satisfactory; 4, high satisfactory; 5, outstanding) [16]. The other instructor who was also blinded to the use of e-learning reviewed the performance and gave the scores through video recordings that the resident’s face was covered post-processing. The instructor at the scene was responsible for the feedback.

(3) From the 8^th^ to the 30^th^ day: Each resident had 15 working shifts and evaluated 10–15 non-critical patients per shift. The resident performed the FAST at her/his discretion and all examinations were written in a standard report form including indication, sonographic findings, sonographic diagnosis, and management.
(4) On the 30^th^ day: The FAST examinations that the PGY-1 residents performed were collected. The US images were reviewed and scored by two instructors independently who were not involved in training and blinded to the use of e-learning, using a 5-point Likert scale (1, no recognizable structures; 2, minimally recognizable structures but insufficient for diagnosis; 3, minimal criteria met for diagnosis, recognizable structures but with some technical flaws; 4, all structures imaged well and diagnosis easily supported; 5, all structures with excellent image quality and diagnosis completely supported) [17].

The instructors reviewed the electronic medical records and made the ‘final diagnosis’ (the existence of fluid at the Morison’s pouch, splenorenal recess, the pelvis, pericardial cavity, pleural cavity, or pneumothorax or not). In case of disagreement, a third expert physician reviewed the medical records and adjudicated the case. Diagnostic accuracy was defined as the agreement between the sonographic diagnosis and the final diagnosis.

The FAST examinations performed by the PGY-2 residents during the same month were also collected on the 30^th^ day. The number of working shifts for PGY-2 was the same as the PGY-1. The PGY-2 received bedside one-to-one teaching in random cases by the attending physicians, a traditional method for US education, before the study. The PGY-2 did not have the authorization to access e-learning. The image quality was scored and the diagnostic accuracy was defined using the aforementioned principles.

### E-learning

**Figure 1.**
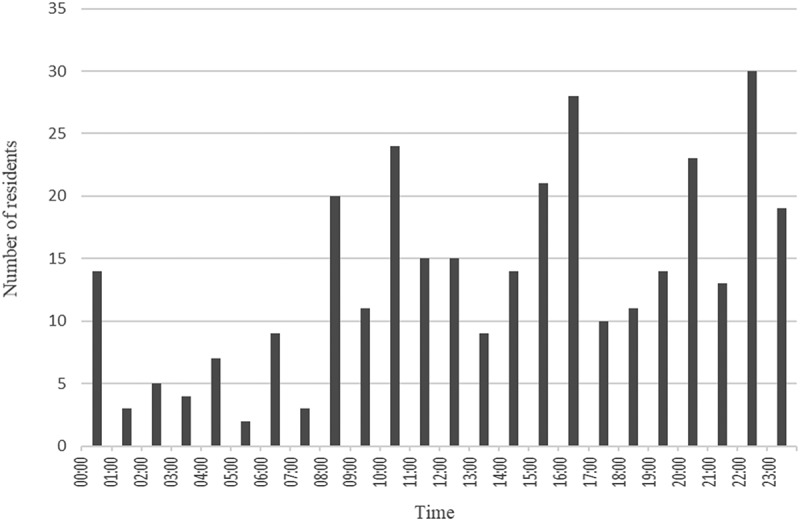
The user interfaces at stage 3 of the e-learning platform.

The e-learning involved 5 stages [15]. The learning process was irreversible among different stages (i.e., the trainee could not return from Stage 3 to Stage 2) and between steps within a stage. Stage 1 was Pretest Stage with multiple-choice questions. Stage 2 was the Teaching Stage where the learners learn the core knowledge. Stage 3 was the Self-Learning Stage to recognize at least 20 US images with interactive image interpretation (Figure 1). Stage 4 was the Self-Testing Stage to evaluate the learning performance in US image recognition. Stage 5 was the Case-Simulation Stage to learn clinical judgments. The user activities to access the different stages and time spent in each stage were automatically recorded. The satisfaction survey was performed before the user logged out (Supplementary file 2).

### Selection of participants

The PGY-1 residents receiving training and performing clinical FAST examinations were included. Those receiving training but not performing any sonographic examinations were excluded. The PGY-2 during the same training month was also included.

### Data collection

Age, sex, self-reported prior US and e-learning experience, and clinical FAST examinations of each PGY-1 and PGY-2 were obtained. The OSCE scores of the PGY-1 resident were obtained. The user activity in the e-learning platform including time spent by each user during his/her first access to the e-learning was collected. The kind of device that the user used to access the e-learning was also collected.

### Outcomes

The clinical FAST performance including the number, imaging quality, and diagnostic accuracy of the FAST examinations between e-learning PGY-1 users, non-users, and the PGY-2 was compared.

### Statistical analysis

The SAS software (SAS 9.4, Cary, North Carolina, USA) was deployed. Initially, we used the Shapiro-Wilk test for the normality of continuous data. If they were not normally distributed, they were expressed in medians and interquartile ranges (IQRs) and examined using Wilcoxon’s rank-sum test. The categorical variables were presented with numbers and proportions and analyzed by a Chi-square test and ANOVA. We used the Intra-Class Correlation (ICC) with the 95% confidence intervals (CIs) to assess inter-rater reliability for the OSCE scores, image quality scores, and diagnostic accuracy.

We applied the linear regression models to identify the factors associated with the FAST performance of the PGY-1. The covariates included age, sex, prior US experience, OSCE score, and the use of e-learning. The polytomous regression models were applied to investigate the factors associated with the OSCE scores. The covariates included age, sex, prior experience, using e-learning before the curriculum, and the accessing device. A p-value of less than 0.05 was considered statistically significant.

## Results

### The characteristics of the participants

During the study period, there were 239 PGY-1 participants, however, 69 residents were excluded due to not performing any FAST examinations.

After the examination of normality, age, prior US experience, OSCE global rating score, the number, imaging quality, and diagnostic accuracy of FAST examinations, the time spent in different stages at the first access, and satisfaction (all p < 0.0001) were not normally distributed and presented with medians and IQRs. The basic information of the PGY-1 and PGY-2 residents was shown in Table 1. All of PGY-1 and PGY-2 reported no structured e-learning experience before. The majority of the PGY-1 were novice sonographers. By contrast, all PGY-2 residents performed more FAST examinations of a median of 26 scans (IQR, 21–32 scans). A total of 736 clinical FAST examinations performed by the PGY-1 and 134 examinations performed by the PGY-2 were collected.

**Table 1.** The characteristics of the post-graduate year (PGY) residents.

Characteristics	PGY-1^†^ using e-learning^‡^ (n = 70)	PGY-1 without using e-learning (n = 100)	PGY-2 (n = 53)	p-value
Age, years*	26 [26]	26 [26]	27 [27]	
Male, n (%)	45 (64%)	73 (73%)	37 (70%)	
Prior PoCUS^†^ experience, scans*	4 [2–5]^a^	3 [2–5]^b^	26 (21–32)^ab^	<0.0001
OSCE^†^ global rating score*	4 [4,5]	4 [4]	-	<0.0001^§^
Sonographic examinations*				
Number*	4 [2–6]^c^	3 [1–5]^d^	2 [1–3]^cd^	0.0018
Imaging quality*	3 (3–3.2)^ef^	3 (2.9–3)^f^	3 (2.7–3)^e^	0.0185
Diagnostic accuracy, %*	100 (100)	100 (100)	100 (100)	0.075

*Presented as median (interquartile range, IQR).

^†^PoCUS, point-of-care ultrasound; OSCE, Objective Structured Clinical Examination; PGY-1, the first postgraduate year.

^‡^Using stage 3 in the e-learning platform.

^§^Compared between the PGYs with and without e-learning.

^a^p<0.0001.

^b^p<0.0001.

^c^p = 0.0004.

^d^p = 0.0074.

^e^p = 0.044.

^f^p = 0.015.

For inter-rater reliability, there is a good ICC coefficient of 0.85 (95% CI, 0.69–0.95) for the OSCE score, 0.83 (95% CI, 0.62–0.95) for the image quality, and 0.90 (95% CI, 0.86–0.93) for diagnostic accuracy between the two instructors.

### E-learning users

**Figure 2.**
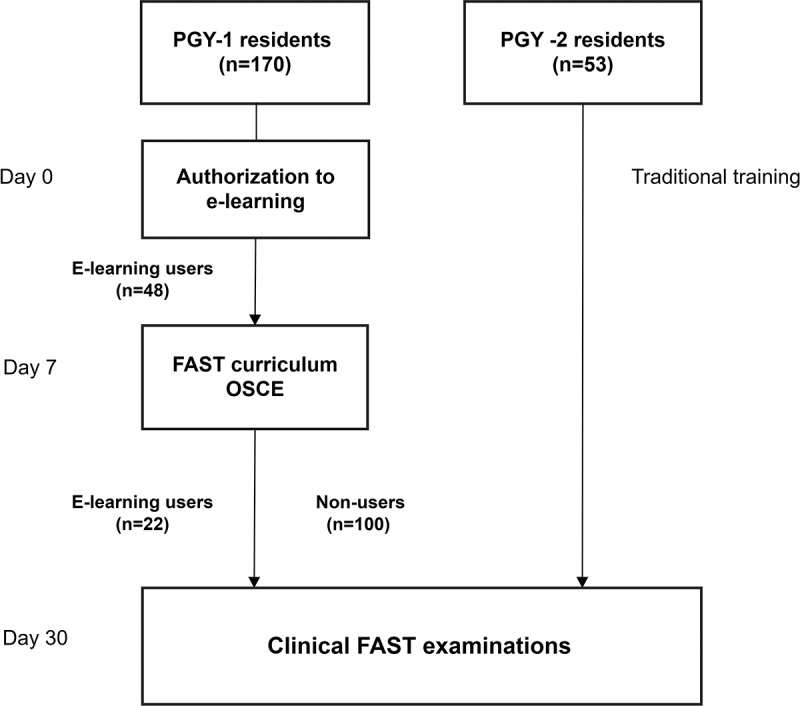
The study diagram. PGY, the post-graduate year; OSCE, objective structured clinical examination; FAST, focused assessment of sonography for trauma.

**Figure 3.**
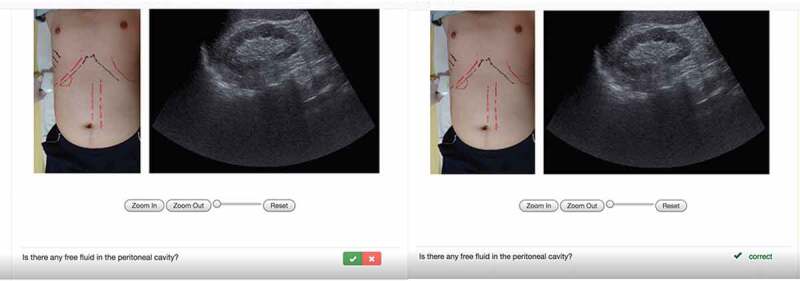
The time of day that the users had the first access to log into the e-learning platform.

Seventy PGY-1 used e-learning (Figure 2) with 324 access and median access of 3 times (IQR, 1–6 times). All 70 residents finished stages 1–3 at the first access. The median time spent was 13.2 mins (IQR, 6.5–21.1 mins) at the first access. The user activity and time spent on the first access to different stages of the e-learning are listed in Table 2. The residents spent a longer duration in the Pretest Stage (Stage 1) and the Teaching Stage (Stage 2). It took a median of 3 minutes to finish the Self-Learning Stage (Stage 3) to recognize at least 20 US images. There was no correlation between the time spent and the number (p = 0.382), imaging quality (p = 0.390), and accuracy (p = 0.759) of FAST examinations. Most of the users used desktops or laptops to access the e-learning (308 access, 95%). User activity, as measured by time distribution, is displayed in Figure 3.

**Table 2.** The user activity and time spent at the first access in different stages of the e-learning platform.

Stages	Accessed residents (n)	Time spent (min)*
Stage 1, Pretest Stage	70	6.5 (4.1–8.2)
Stage 2, Teaching Stage	70	5.6 (3.0–11.7)
Stage 3, Self-Learning Stage	70	3.3 (2.4–4.6)
Stage 4, Self-Testing Stage	25	1.3 (1.0–1.8)
Stage 5, Case-Simulation Stage	21	4.1 (3.8–5.0)

*Presented as median (interquartile range, IQR)

The users were satisfied with the platform design (median, 4.5, IQR, 4–5), content (median, 5, IQR, 4–5), the quality of the image (median, 5, IQR, 4–5), and the designated training course (median, 5, IQR, 4–5).

### The impact of e-learning on clinical FAST examinations

Compared with the PGY-2, the PGY-1 residents (e-learning users and non-users) performed more FAST examinations during clinical practice (Table 1). The e-learning users had a trend to do more FAST than non-users although the results were non-significant. Also, the e-learning users had better image quality than non-users and PGY-2. There were no significant differences in the diagnostic accuracy between the PGY-1 and PGY-2.

We applied the linear regression models to investigate the factors associated with the image quality of the FAST examinations performed by the PGY-1. After adjusting age, sex, prior US experience, and OSCE scores, using e-learning remained significant to be associated with better image quality (coefficient, 0.21 ± 0.09, p = 0.014).

### The impact of e-learning on the OSCE scores of the PGY-1

The e-learning PGY-1 users had better OSCE performance than non-users (Table 1). To investigate the effect of the timing to use the e-learning, the 70 residents were categorized based on accessing the e-learning before and after the curriculum (Table 3). Forty-eight residents with prior access to the e-learning before the curriculum had better performance in the OSCE than those accessing the e-learning after the curriculum (median [IQR], 5 [4–5] vs. 4 [4], p = 0.001, Supplementary file 3).

**Table 3.** The characteristics of the first-year post-graduate year (PGY-1) residents using the e-learning before and after the curriculum.

Characteristics	Before the curriculum (n = 48)	After the curriculum (n = 22)	p-value
Male, n (%)	31 (65%)	14 (64%)	
Prior US* experience, n (%)			
≤10 scans	44 (92%)	22 (100%)	
> 10 scans	4 (8%)	0	
OSCE global rating score^†^	5 [4,5]	4 [4]	0.001
Sonographic examinations^†^			
Number^†^	4 [1–6]	3 [2–5]	
Imaging quality^†^	3 (3–3.3)	3 [3]	
Diagnostic accuracy, %^†^	100 (96.7–100)	100 (100)	

*US, ultrasonography; OSCE, Objective Structured Clinical Examination.

^†^Presented as median (interquartile ranges, IQRs).

However, no significant differences existed in the numbers, image quality, and accuracy of the FAST examinations. After adjusting age, sex, prior PoCUS experience, and the route of access to the e-learning, using the e-learning before the curriculum was positively associated with the OSCE scores (coefficient, 0.56 ± 0.09, p < 0.0001).

## Discussion

The study investigates the effect of e-learning on the clinical US performance of PGY-1 residents. The results demonstrated the feasibility of integrating an e-learning program into PoCUS training for novice sonographers in a busy environment. The PGY-1 could use e-learning at any time. The e-learning users had a comparative US performance with the PGY-2 and even better image acquisition than PGY-2 although the PGY-2 had more PoCUS experience. Also, they had better OSCE performance than the PGY-1 non-users. Moreover, the users had high satisfaction with e-learning.

Physicians are becoming increasingly interested in web-based education with the rapid advances in information and communication technologies. The current trainees have a strong propensity for the use of web- and social media-based curricula [10,18]. The use of e-learning would compensate for the time barrier and provide flexibility in learning. Further, the relevance of e-learning in medicine is enormously increasing during the COVID-19 pandemic [19].

Concerning the use of e-learning when acquiring theoretical or practical skills, previous literature proves the value of e-learning in learning theory, however, the result regarding e-learning and practical skills are limited [20,21]. Previous studies frequently used an online survey, questionnaires, or OSCE to evaluate the efficacy of e-learning [14,22–24]. However, few studies have evaluated whether e-learning prepares trainees effectively for clinical performance [25,26]. To the best of our knowledge, this is the first study to evaluate the efficacy of e-learning in clinical performance. An emergency resident receiving traditional training has to perform at least 50 sonographic examinations for the FAST application to have recognizable image acquisition [6]. Our results showed the use of e-learning was associated with the acceleration of US learning and skill transfer in clinical US performance. E-learning could shorten the learning time of the focused PoCUS application.

The optimal timing for the use of e-learning was still uncertain. Previous randomized studies showed comparable outcomes in knowledge and skills after web-based learning was introduced before or after the hands-on training [23,27]. The results demonstrated that using e-learning before the curriculum had a significant impact on the OSCE scores. It indicated that previewing the learning materials played an important role in better understanding sonographic techniques and standard images, resulting in a better OSCE performance. However, clinical US performance was similar between the users before and after the curriculum, in agreement with those in previous studies [23,27].

The social cognitive theory has proposed that people acquire new skills by observing others and modeling [28]. In this study, all of the PGY-1 were novices. The learning process of e-learning could be one part of skill modeling, refining, and integration. Therefore, the PGY-1 residents using the e-learning had better image acquisition than non-users and the PGY-2.

The key points of e-learning include content management, delivery, and standardization [9]. US training involves 3 essential components: knowledge gain, development of psychomotor skills, and visual perception for image acquisition, interpretation, and integration into medical decision-making [7]. The development of the e-learning was based on the aforementioned concept: knowledge dissemination at Stage 2, image recognition at Stage 3 accompanied by US probe position, followed by the clinical scenario at Stage 5. Current online resources for US education such as lectures, still images, or videos provided only unilateral information to users [12]. Our e-learning preserved the advantages of traditional US training, including the interaction between teachers and learners, immediate feedback, and clinical decision-making. Also, the user activity demonstrated that e-learning could occur at any time and the users could review materials at desired pace and frequency, giving flexibility.

A previous study reported that most current learners preferred shorter web-based lectures with an ideal duration of approximately 10 to 15 minutes [29]. The user through Stages 1 to 3 in the e-learning needed approximately 13 minutes. That could be explained that most of the users stopped at Stage 3.

Better image acquisition is observed in the PGY-1 users. The results were corresponding to the most frequently used content, Stage 3 in e-learning. In Stage 3, visual stimulation of probe position and standard images of the FAST is repeatedly presented with timely feedback. The users could practice again and again visually, continue the reflection, and gradually refine psychomotor skills. However, the diagnostic accuracy was similar between PGY-1 users and non-users. The results seemed to imply that using e-learning had trivial improvements in image interpretation. Nevertheless, the median accuracy is 100% in this study and the improvements would not make the statistical results significant. On the other aspect, every resident evaluated 200–250 patients in total during the training month. The median number of scanning cases was 3. It indicated that the residents used PoCUS in highly selected, maximal self-confident cases, resulting in 100% accuracy. Moreover, the PGY-1 non-users had similar diagnostic accuracy to the PGY-2, implying the positive effect of the focused curriculum.

Furthermore, there were different ways to access e-learning. Although smartphones or tablets enabled users to learn at any place and time [30], in this study, most of the users were accustomed to using desktops or laptops at fixed locations. It would be not surprising that the residents, novice sonographers, learned a new PoCUS skill seriously. Also, the resolution of the images would be much better on desktops or laptops.

This study had limitations. First, there were statistically significant differences in OSCE scores, the number, and imaging quality of FAST examinations between the PGY1 users and non-users. The diagnostic accuracy of FAST examinations was similar between the two groups. The clinical relevance was wondered. However, we investigated the effect of e-learning on US education and performance. We found e-learning users had comparable performance with the PGY-2. It implied e-learning enhanced the learning of US imaging. Second, we measured the short-term effect on clinical FAST performance after using e-learning. The long-term sustained effect and further utilization of e-learning must be assessed, however, the data was hard to collect because most of the PGY-1 and PGY-2 would not return to the ED again during their training course. Third, the participants were not randomized to use the e-learning. The previous study showed highly motivated and self-disciplined students, e-learning could be as effective as traditional teaching [31]. Hawthorne effect would exist in our users. However, the use of e-learning remained significantly to be associated with better clinical performance after adjusting for possible confounders. The structured e-learning could help the novices perform comparably with the PGY-2. Fourth, this study only evaluated the FAST performance. However, FAST was the fundamental application of PoCUS, being used not only in trauma victims but in non-trauma patients. It was very suitable as the teaching material during a short-course, focused US training curriculum. Last, this study was conducted at an academic hospital with active US training. Someone would wonder if the experience could not be extrapolated to other specialties. However, the applications of PoCUS are rapidly growing in recent decades. Our results provide evidence that e-learning has a positive impact on focused PoCUS training, especially for novice sonographers in a busy environment.

In conclusion, e-learning has a positive effect on US learning for novices. The PGY-1 users had comparable performance with the PGY-2 and even better image acquisition although the PGY-2 had more PoCUS experience. However, the long-term sustained effect regarding e-learning and clinical relevance should be further investigated.

## Supplementary Material

Supplemental Material

## Data Availability

All data generated or analyzed during this study are included in this published article.

## List of abbreviations

Point-of-care ultrasound (PoCUS);

Focused assessment of sonography for trauma (FAST);

Ultrasound (US);

Electronic learning (e-learning);

First post-graduate year (PGY-1);

Second post-graduate year (PGY-2);

Emergency Department (ED);

Objective structured clinical examination (OSCE);

Interquartile range (IQR);

Intra-Class Correlation (ICC);

Confidence intervals (CIs).
