# Supplementary material for: The effect of e-learning on point-of-care ultrasound education in novices
Source: Med Educ Online. 2022 Nov 26;28(1):2152522. doi: 10.1080/10872981.2022.2152522 (PMC9707377; doi:10.1080/10872981.2022.2152522)
Supplement: Supplemental Material [file ZMEO_A_2152522_SM3995.zip › Supplementary/Supplementary file_1_FAST_OSCE_anonymus.docx]

**Point-of-Care Ultrasound Assessment Form**

Application: Focused assessment with sonography for trauma(FAST)

Scenario: A 70-year-old man suffered from an abdominal contusion following an accidental fall when walking.

Name: _______________ Time: __________________

| Item | Checklist | None | Partial | Complete | Note |
| --- | --- | --- | --- | --- | --- |
| 01 | Self-introduction |  |  |  |  |
| 02 | Patient identification |  |  |  |  |
| 03 | Introduction to the patient for the examination |  |  |  |  |
| 04 | Probe choosing |  |  |  |  |
| 05 | Morison’s pouch： | | | | |
|  | Liver identification |  |  |  |  |
|  | Kidney identification |  |  |  |  |
|  | Morrison pouch |  |  |  |  |
| 06 | Splenorenal recess： | | | | |
|  | Spleen identification |  |  |  |  |
|  | Kidney identification |  |  |  |  |
|  | Spleno-renal recess |  |  |  |  |
| 07 | Pelvic view： | | | | |
|  | Urinary bladder (transverse) |  |  |  |  |
|  | Urinary bladder (longitudinal) |  |  |  |  |
|  | Rectovesical space |  |  |  |  |
| 08 | Subxiphoid view： | | | | |
|  | Heart |  |  |  | Partial: 4 chambers partially identified. |
| 09 | Explain the results to the patient |  |  |  |  |
| 10 | Freeze and clean the probe |  |  |  |  |

Global assessment：

| Likert’s 5-point scale | 1 | 2 | 3 | 4 | 5 |
| --- | --- | --- | --- | --- | --- |
|  |  |  |  |  |  |

Comments: Assessor：______________
