# Supplementary material for: The effect of e-learning on point-of-care ultrasound education in novices
Source: Med Educ Online. 2022 Nov 26;28(1):2152522. doi: 10.1080/10872981.2022.2152522 (PMC9707377; doi:10.1080/10872981.2022.2152522)
Supplement: Supplemental Material [file ZMEO_A_2152522_SM3995.zip › Supplementary/Supplementary file_2_satisfaction.docx]

Dear learners, we thank you for your support and cooperation. To serve you better in the future, please fill in the following. Thank you!

1. Are you satisfied with our web design?

(1) Very dissatisfied.

(2) Dissatisfied.

(3) Just so so.

(4) Satisfied.

(5) Very satisfied.

2. Are you satisfied with our content?

(1) Very dissatisfied.

(2) Dissatisfied.

(3) Just so so.

(4) Satisfied.

(5) Very satisfied.

3. Are you satisfied with the quality of the image?

(1) Very dissatisfied.

(2) Dissatisfied.

(3) Just so so.

(4) Satisfied.

(5) Very satisfied.

4. Are you satisfied with this course that can help you in your career?

(1) Very dissatisfied.

(2) Dissatisfied.

(3) Just so so.

(4) Satisfied.

(5) Very satisfied.

5. Are you satisfied with the training course at all?

(1) Very dissatisfied.

(2) Dissatisfied.

(3) Just so so.

(4) Satisfied.

(5) Very satisfied.

6. Your suggestions?
