# Supplementary figures and images for: The effect of e-learning on point-of-care ultrasound education in novices
Source: Med Educ Online. 2022 Nov 26;28(1):2152522. doi: 10.1080/10872981.2022.2152522 (PMC9707377; doi:10.1080/10872981.2022.2152522)

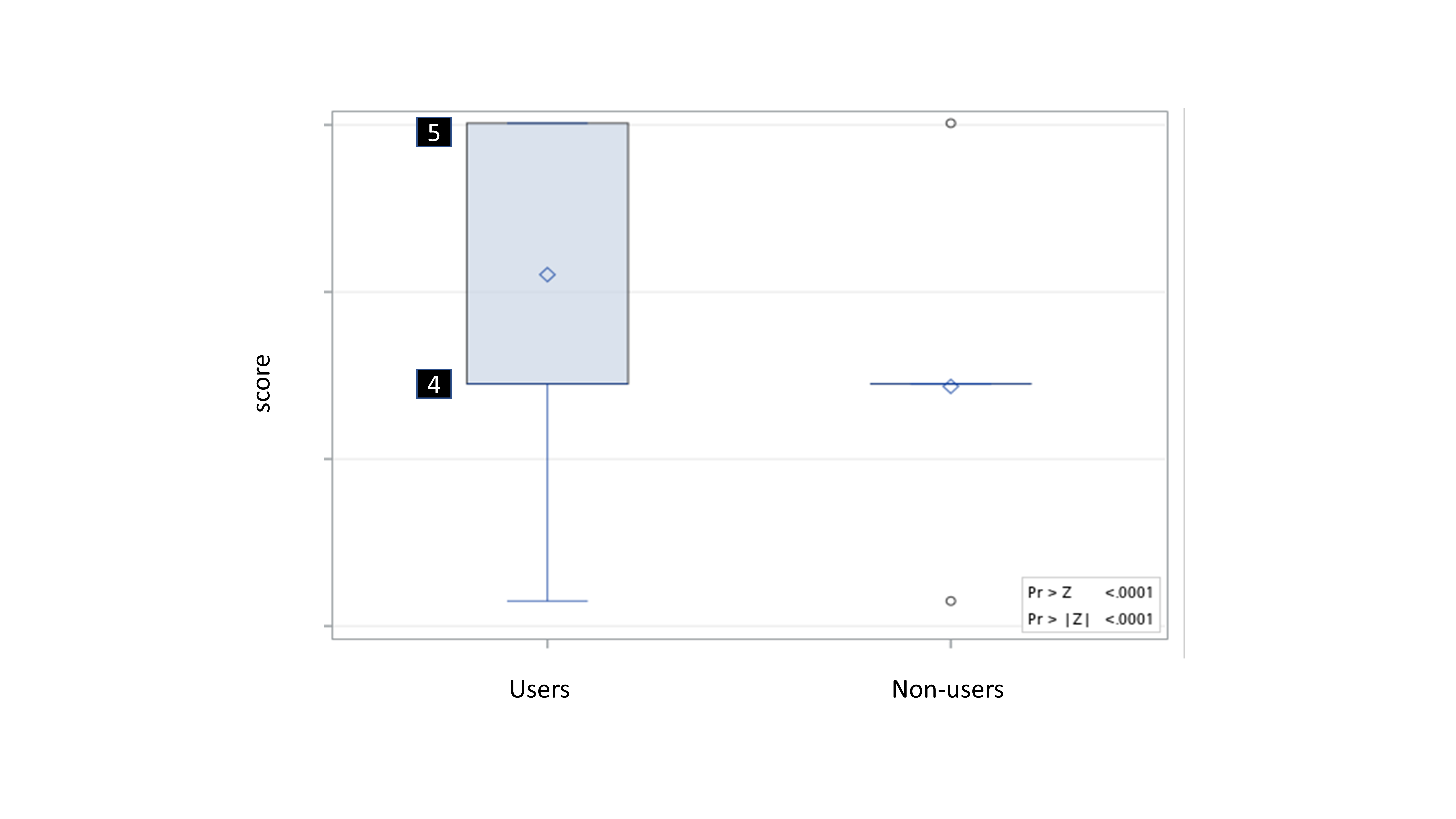

Supplement: Supplemental Material [file ZMEO_A_2152522_SM3995.zip › Supplementary/Supplementary file 3_OSCE_users_nonusers.tif]
